# High diagnostic accuracy of automated rapid Strep A test reduces antibiotic prescriptions for children in the United Arab Emirates

**DOI:** 10.1186/s12887-021-02516-3

**Published:** 2021-01-25

**Authors:** Salama Bin Hendi, Zainab A. Malik, Amar Hassan Khamis, Fadil Y. A. Al-Najjar

**Affiliations:** 1 College of Medicine, Mohamed Bin Rashid University of Medicine and Health Sciences, Dubai Healthcare City, Building 14, Dubai, 505005 United Arab Emirates; 2 Department of Pediatrics, Mediclinic City Hospital. Dubai Healthcare City, Building 37, Dubai, 505004 United Arab Emirates; 3 Pediatric Infectious Diseases, Mediclinic City Hospital. Dubai Healthcare City, Building 37, Dubai, 505004 United Arab Emirates; 4 Department of Biostatistics, Mohamed Bin Rashid University of Medicine and Health Sciences. Dubai Healthcare City, Building 14, Dubai, 505005 United Arab Emirates

**Keywords:** Pharyngitis, Children, Rapid strep, Throat culture, Diagnostic accuracy, Antibiotics, *Streptococcus pyogenes*

## Abstract

**Background:**

Diagnosis of Group A *Streptococcus* (GAS) pharyngitis in children is hindered by variable sensitivity of clinical criteria and rapid Strep A tests (SAT), resulting in reliance on throat cultures as the gold standard for diagnosis. Delays while awaiting culture reports result in unnecessary antibiotic prescriptions among children, contributing to the spread of antimicrobial resistance (AMR).

**Methods:**

Diagnostic accuracy study of an automated SAT (A-SAT) in children up to 16 years of age presenting to an emergency room with signs and symptoms of pharyngitis between March and June 2019. Paired throat swabs for A-SAT and culture were collected. Sensitivity, specificity, positive predictive value (PPV) and negative predictive value (NPV) for A-SAT were calculated.

**Results:**

Two hundred and ninety-one children were included in this study. 168 (57.7%) were boys and the mean age was 4.2 years. A-SAT was positive in 94 (32.3%) and throat culture was positive in 90 (30.9%) children. A-SAT and throat culture results showed a high level of consistency in our cohort. Only 6 (2%) children had inconsistent results, demonstrating that the A-SAT has a high sensitivity (98.9%), specificity (97.5%), PPV (94.7%) and NPV (99.5%) for the diagnosis of GAS pharyngitis in children. Only 92 (32%) children were prescribed antibiotics while the vast majority (68%) were not.

**Conclusions:**

A-SAT is a quick and reliable test with diagnostic accuracy comparable to throat culture. Its widespread clinical use can help limit antibiotic prescriptions to children presenting with pharyngitis, thus limiting the spread of AMR.

## Background

Antimicrobial resistance (AMR) is a rapidly-growing global health threat [1]. Infections caused by antibiotic-resistant pathogens increase the burden of both healthcare-associated and community-acquired infections, leading to increased healthcare utilization and high mortality [2, 3]. Inappropriate antibiotic use in community and healthcare settings is a major driver of antibiotic resistance worldwide [4].

Recent estimates suggest that over 700,000 people die annually from antibiotic-resistant infections globally [5]. International agencies have warned that in the absence of a coordinated global response to this epidemic, the death toll from drug-resistant infections could climb to 10 million annually, forcing 24 million people globally into extreme poverty (World Health Organization. New report calls for urgent action to avert antimicrobial resistance crisis. https://www.who.int/news-room/detail/29-04-2019-new-report-calls-for-urgent-action-to-avert-antimicrobial-resistance-crisis, 2019).

Children are often prescribed antibiotics for potential streptococcal pharyngitis when in fact their signs and symptoms are suggestive of viral upper respiratory tract infections (URTIs) [6, 7]. It has been demonstrated that antibiotic prescribing practices vary by country [8] and by provider specialty [9]. Inappropriate antibiotic use results in increased rates of AMR and increased healthcare utilization due to adverse drug reactions [10, 11].

Acute pharyngitis due to *Streptococcus pyogenes* or group A *β-hemolytic streptococcus* (GAS) is diagnosed in up to 37% of children with pharyngitis [12] and requires antibiotic treatment. This has been traditionally thought of as an infection of school-age children; however, a recent meta-analysis estimated the prevalence of GAS pharyngitis in children under 5 years at 25%, supporting diagnostic testing in this age group when clinically indicated [13].

GAS is a leading global pathogen of infectious diseases [1]. The spectrum of GAS disease extends from superficial infections (pharyngitis, impetigo) to invasive disease (abscesses, cellulitis, sepsis), toxin-mediated manifestations (scarlet fever, toxic shock syndrome, necrotizing fasciitis) and autoimmune sequelae (acute rheumatic fever [ARF], poststreptococcal glomerulonephritis and rheumatic heart disease [RHD]). The most frequent manifestation of GAS infection is pharyngitis, with over 600 million new cases diagnosed per year and complications from invasive GAS disease resulting in 500,000 deaths globally [14].

Timely and targeted antibiotic treatment of GAS infections constitutes the backbone of prevention of complications [15]. This has resulted in a reduction in the burden of RHD in high-income countries; however, high rates of disease persist in low-income countries compounded by lack of healthcare access and unavailability of adequate diagnostic testing [16].

Clinical features and history do not reliably discriminate between GAS and viral pharyngitis, especially in children [17, 18]. International guidelines recommend throat swabs for rapid antigen testing and GAS culture for patients with acute pharyngitis [19] to rapidly detect and target antibiotic treatment for those with GAS infection. The main rationale for antibiotic therapy for GAS pharyngitis is to prevent the development of ARF, the most common cause of acquired heart disease in children. RHD constitutes a substantial burden of chronic disability and death among adolescents and young adults [15, 20].

Throat cultures are considered the gold standard for diagnosis of GAS pharyngitis. A recent systematic review demonstrated an 85% diagnostic sensitivity of SAT among children [21]. However, this sensitivity is suboptimal for rapid assays to be considered stand-alone diagnostic tests, and concurrent back-up throat cultures are required for definitive diagnosis of GAS in children with a negative SAT.

We have previously reported a high diagnostic accuracy of QuickVue® Dipstick Strep A rapid test in a paediatric outpatient clinic setting where high-quality swabs were taken by the attending paediatrician [22]. A similar study in the UAE demonstrated a similarly high diagnostic accuracy of Diaquick Strep A Test [23]. Other studies have also demonstrated a high test sensitivity when throat swabs for SAT are collected by a paediatrician [24–26]. However, these studies’ findings are not generalizable to a “real-world” setting where samples are often taken by nurses or non-paediatric physicians, necessitating the need for back-up throat cultures in routine practice to minimize false-negative diagnoses of GAS pharyngitis that would increase the risk of post-infectious sequelae.

In our current study, we evaluated the use of a newer automated rapid streptococcal antigen test system (A-SAT) in children presenting with pharyngitis. Standard F strep A antigen test (SD Biosensor, Republic of Korea) is a fluorescence immunoassay with a manufacturer-reported test sensitivity of 93.3% and specificity of 95% compared to throat culture. We designed a study to determine its diagnostic accuracy in a “real world” scenario in an emergency room where samples are taken by non-paediatric nurses, and to determine its impact on antibiotic prescribing practices in children presenting with pharyngitis.

## Methods

This was a single-gated diagnostic accuracy study with prospective data collection conducted from March to June 2019 in the emirate of Dubai, United Arab Emirates. Children up to 16 years of age presenting to the emergency department of a multidisciplinary university-affiliated hospital with signs and symptoms of pharyngitis were included in our study. We excluded children if they were older or if both the automated rapid streptococcal antigen test (A-SAT) and throat culture were not done. Two-hundred and ninety-one children were included in our study.

All subjects were evaluated and screened for study eligibility by the emergency room (ER) physician. Symptom recording was standardized and all history and physical exam findings were recorded into the hospital’s electronic medical record (EMR) by ER physicians. Throat swabs were collected by ER nurses. Patients who had paired throat swabs collected for A-SAT and culture were included in our analysis.

The paired samples were collected with a sterile dry dual red top swab and stored in a clear and dry plastic tube. The samples were transported to the Pathology and Laboratory Department at the Mediclinic City Hospital as soon as possible after collection for testing. This laboratory runs 24 h and is accredited by the College of American Pathologists (CAP), Joint Commission International (JCI) and holds the ISO-15189 certification. Throat swabs for culture were inoculated as described previously [22].

The A-SAT is an automated fluorescence immunoassay used to detect streptococcal antigen in throat specimens from patients with clinical symptoms. Bacterial antigen is extracted from the patient’s swab by the laboratory technician and placed into the sample well of the test device for analysis. Pre-programmed algorithms of the automated device display test results on the device’s screen. The measured fluorescence signal is numerically represented by a cut-off index value (COI). A COI greater than or equal to 1.0 is interpreted as positive, while a COI of less than 1.0 is interpreted as a negative test.

The automated fluorescence immunoassay takes 10–15 min to perform. However, there is a delay of 30–60 min between collecting the sample and results being uploaded on the hospital’s EMR. Once results are available, the ER physician usually makes antibiotic prescription decisions. Some parents request a prescription and leave pending the A-SAT results. Once results are available, the ER doctor calls and informs the parents and provides guidance on whether or not antibiotics should be started.

In all cases, parents signed a consent form at the time of their child’s hospital registration. Collection of throat swabs was done as a part of the child’s healthcare process, which is covered by the above general consent. Hence, no separate consent was obtained from parents at the time of sample collection.

This study was reviewed by the Institutional Review Board (IRB) of Mohammed Bin Rashid University of Medicine and Health Sciences (MBRU) Student Research Projects (SRP) committee (MBRU-IRB-SRP-104-2017). The data was analyzed using the Statistical Package for Social Sciences (SPSS) 24 software. Our study followed the STARD reporting protocol. Since this was a time-frame study, no power or sample size calculations were required.

## Results

Two hundred and ninety-one children were enrolled in our study. Their mean age was 4.2 years, ranging from 5 months to 16 years. Only 65 (22%) children were Emiratis, and the rest were expatriates representing various ethnicities all over the world. 187 (64.7%) children were under the age of 5 years and 168 (57.7%) were male. Most children had a history of fever (93.4%), and ten (3.4%) reported receiving a dose of antibiotics prior to presentation.

**Fig. 1:**
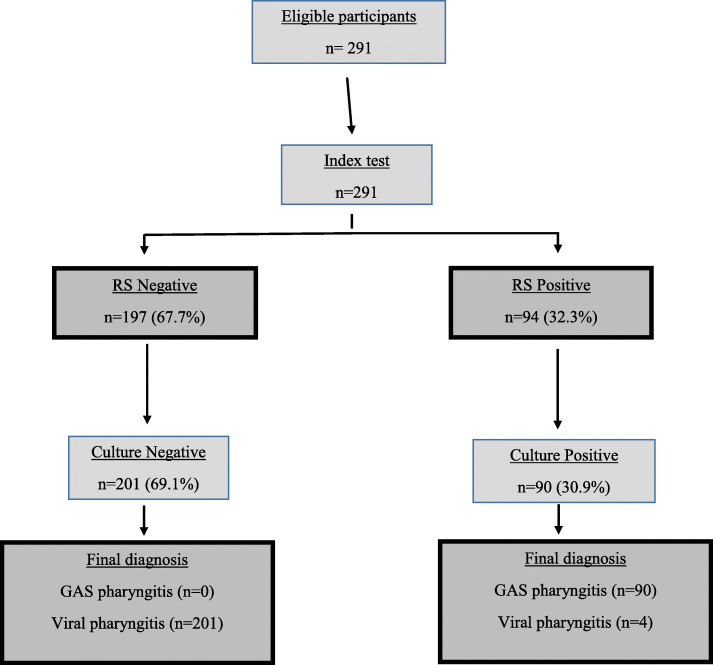
Flow of participants through study in a STARD prototype

Overall, A-SAT was positive in 94 (32.3%) and negative in 197 (67.6%) patients, while throat culture was positive in 90 (30.9%) and negative in 201 (69%) (Fig. 1). There was inconsistency between A-SAT and culture results in six children. A-SAT only missed one child with GAS pharyngitis who tested positive on throat culture (Table 1).

**Table 1:** Automated rapid streptococcal antigen test (A-SAT) and throat culture results (*n* = 291)

		**A-SAT**		
		Negative	Positive	**Total**
**Culture**	Negative	196	5	201
	Positive	1	89	90
**Total**		197	94	291

The sensitivity and specificity of A-SAT in the identification of GAS pharyngitis were 98.9% (95% CI 98, 100%) and 97.5% (95% CI 95.7, 99.3%) respectively. A-SAT had a PPV of 94.7% (95% CI 92.1, 97.3%) and NPV of 99.5% (95% CI 98.7, 100%) (Table 2).

**Table 2:** Clinical performance of automated rapid Strep A test (A-SAT) when compared to throat culture for the detection of Group A *Streptococcus* pharyngitis (*n* = 291)

	A-SAT
**Sensitivity (95% CI)**	98.9% (98–100)
**Specificity (95% CI)**	97.5% (95.7–99.3)
**PPV (95% CI)**	94.7% (92.1–97.3)
**NPV (95% CI)**	99.5% (98.7–100)

Out of a potential 291 antibiotic prescriptions, only 94 children (32%) were prescribed oral antibiotics for GAS pharyngitis, while the vast majority (68%) were not. For one child with negative A-SAT and positive throat culture, parents were informed of the result over the phone and antibiotics were initiated.

## Discussion

This is the first study evaluating the accuracy of A-SAT in the Middle East. We have previously reported a high diagnostic accuracy of rapid streptococcal antigen tests, comparable to throat culture, when performed by an attending pediatrician in the outpatient clinic setting in the United Arab Emirates [22, 23]. Unfortunately, those findings were not generalizable to a “real world” scenario where children are often assessed by general physicians and throat samples are taken by non-pediatric healthcare providers. Studies have reported variation in antigen test performance by inoculum size and provider characteristics, resulting in lower test sensitivity attributable to suboptimal sampling techniques and provider experience in obtaining samples from children [25, 26].

In this study, we have demonstrated a very high diagnostic efficacy of A-SAT, with NPV approaching 100% in children presenting to the ER with signs and symptoms of pharyngitis. This shows that new-generation rapid streptococcal antigen assays that utilize fluorescent immunoassay technology, are robust to variations in sampling technique and perform well in real-world clinical scenarios, making them very useful in providing rapid and accurate diagnosis of GAS pharyngitis in children.

As expected, most children had viral illnesses contributing to their symptoms, as confirmed by negative throat cultures in 69% of our study cohort. Since our study objective was to examine the diagnostic accuracy of A-SAT in comparison to throat culture, we decided to include 10 children who reported receiving antibiotics prior to presentation; all of them had negative A-SAT and throat cultures.

Among our patients, the prevalence of GAS pharyngitis was 30.9%. This is higher than the previously reported prevalence of 14% between 2004 and 2006 [23] and 20.6% between 2016 and 2018 [22] among children in the United Arab Emirates. The increase in reported prevalence in our current study could represent a referral bias of sicker children presenting to the ER rather than to an outpatient clinic. These figures are consistent with the reported prevalence of GAS pharyngitis ranging between 30 and 37% in other pediatric studies [13, 21].

Our study was conducted in the emirate of Dubai in the United Arab Emirates, which is home to people from over 200 countries. Expatriates constitute 80% of the population of Dubai, home to one of the world’s largest percentage of immigrants. Our patient cohort was highly representative of Dubai’s population mix, with expatriates constituting around 78% of the study population. Hence, our study findings are likely generalizable to children from various ethnicities around the world.

The current recommendations of throat cultures as a confirmatory test on patients with a negative rapid test [19] is not achievable in most low-income countries due to the prohibitive cost. Ehrlich et al. have reported that follow-up throat cultures for negative rapid tests would detect 21 additional cases of rheumatic heart disease at a societal cost of an additional $8 million per case prevented in the United States [27]. Among South African children, obtaining throat cultures in all children presenting with pharyngitis as a primary prevention strategy for RHD is prohibitively expensive; treating all such children with intramuscular penicillin is the most cost-effective strategy [15] but risks the rapid development of AMR. The high sensitivity and NPV of A-SAT in our study suggests that this can be used as a stand-alone test, without the routine need for confirmatory throat cultures, striking a balance between reducing costs and preventing excessive use of antibiotics especially in resource-lacking countries. However, it needs further review before this strategy can be adopted into routine clinical practice.

Utilizing A-SAT testing provided rapid and accurate results, resulting in a striking reduction in antibiotic prescriptions among our cohort. The vast majority (*n* = 197, 68%) of children in our study were not prescribed antibiotics for acute pharyngitis. Fewer antibiotic prescriptions result in direct monetary savings, reduction in antibiotic use, and decreased emergence of resistant pathogens in our community. This reduction in antibiotic use also helps to reduce the clinical and economic burden of adverse drug reactions and unnecessary healthcare utilization due to these effects [10, 11].

The main strength of our study was that it was conducted in the busy ER of a tertiary care hospital with a diverse patient population from various ethnicities. This represents a real-world scenario and increases the applicability of our findings to other clinical settings. The automation of A-SAT minimizes false positive results that can result from prolonged incubation times when tests are performed manually. This likely improved the diagnostic accuracy of A-SAT in our study. In addition, all patients underwent A-SAT and throat culture on paired swabs collected simultaneously, hence limiting differential verification bias. Paired swabs also ensured that there was no delay in sample testing, which could lead to over- or underestimation of results. The major limitation of our study is that it represents a convenience sample of patients presenting to the ER of a single multidisciplinary hospital rather than an array of hospitals, hence limiting its generalizability to other clinical settings.

## Conclusions

This is the first study to demonstrate that the diagnostic accuracy of an automated rapid Strep A test among children in an emergency room setting in the Middle East is comparable to that of throat culture. We have demonstrated that the A-SAT is a simple, rapid, and highly reliable test which can help reduce unnecessary antibiotic prescriptions in children presenting with pharyngitis, and hence limit the spread of antimicrobial resistance.

## Data Availability

Data which supports the findings of this study is available from Mediclinic City Hospital. However, restrictions apply to the availability of this data, which was used under license for the current study and is not publicly available. Study data can be made available upon reasonable request from the authors and with permission of Mediclinic City Hospital, Dubai, UAE.

## Abbreviations

GAS: Group A β- *haemolytic Streptococcus*
SAT: Rapid streptococcal A antigen test
AMR: Antimicrobial resistance
A-SAT: Automated rapid streptococcal A antigen test
PPV: Positive predictive value
NPV: Negative predictive value
URTI: Upper respiratory tract infections
ARF: Acute rheumatic fever
RHD: Rheumatic heart disease
ER: Emergency Room
EMR: Electronic medical record
CAP: College of American Pathologists
JCI: Joint Commission International
COI: Cut-off index
IRB: Institutional Review Board
MBRU: Mohammed Bin Rashid University of Medicine and Health Sciences
SRP: Student Research Projects
SPSS: Statistical Package for Social Sciences
